# Induction Effect of Bisphenol A on Gene Expression Involving Hepatic Oxidative Stress in Rat

**DOI:** 10.1155/2016/6298515

**Published:** 2016-04-10

**Authors:** Sohrab Kazemi, Seydeh Narges Mousavi, Fahimeh Aghapour, Boshra Rezaee, Farzin Sadeghi, Ali Akbar Moghadamnia

**Affiliations:** ^1^Cellular and Molecular biology research centre, health research institute, Babol University of Medical Sciences, Babol, Iran; ^2^Department of Pharmacology, Faculty of Medicine, Babol University of Medical Sciences, Babol, Iran; ^3^Department of Microbiology, Faculty of Medicine, Babol University of Medical Sciences, Babol, Iran

## Abstract

*Background and Objective.* Bisphenol A (BPA) is an abundantly used xenoestrogenic chemical which may cause various disorders in body. In the present study, we sought to investigate the effects of various doses of BPA on hepatic oxidative stress-related gene expression in rats.* Methods.* Male Wistar rats weighing 150–200 g were used in this study. Three doses of the BPA (5, 25, and 125 *μ*g/kg) in corn oil were administered as gavage during 35 consecutive days. After the experiment, the rats were expired and the livers were removed and stored at −80°C freezer for RNA extraction.* Findings.* The Real Time PCR showed increased expression of HO-1 in the rats receiving BPA doses compared to the control group. This effect was dose-dependent and higher at doses of 25 and 125 *μ*g/kg than 5 *μ*g/kg of body weight (*p* < 0.05). It was also demonstrated that various doses BPA can increase GADD45B gene expression compared to control group. That expression was significantly dominant in the lowest dose (5 *μ*g/kg) of the BPA (*p* < 0.05). The final body weights (168.0 ± 10.0 gr) in the treatment group [BPA (125 *μ*g/kg)] showed a significant decrease compared to control group (191.60 ± 6.50 gr).* Conclusion.* These findings demonstrate that BPA generated ROS and increased the antioxidant gene expression that causes hepatotoxicity.

## 1. Introduction

Bisphenol A (BPA) is a synthetic xenoestrogenic compound as [1] which is widely used as a key monomer in production of various polycarbonate plastics and epoxy resins, such as food and drink containers, baby bottles, and dental sealants [2].

Although the BPA is not dangerous in polymeric form, it is unstable in acidic and basic solutions and exposure to UV light. Those conditions may convert the polymeric BPA to monomeric forms. In this condition BPA is released into food, beverages, or environment [3]. Due to long-term release of the PBA from food product containers, most individuals in general populations are widely exposed to this substance, according to the fact that every year hundreds of tons of the BPA are released into atmosphere [2]. It is water soluble as reported at high level in marine populations [4]. Many investigations have been conducted to evaluate the toxic effects of the BPA on human health [5].

Several studies have revealed the toxic effect of BPA (even at low doses) on various organs by increasing oxidative stress [6]. The liver is an important organ that has been affected by the BPA through inducing oxidative stress [6–8]. Reactive oxygen species (ROS) such as superoxide, hydroxyl and proxy radicals, and hydrogen peroxide are cytotoxic agents that are able to stimulate oxidative stress by impairment of prooxidant/antioxidant balance [9, 10].

Up to date, so many investigations have demonstrated the impact of the BPA induced oxidative stress on various genes in liver [8, 11]. HO-1 and GADD45B are two genes which their expression can be affected on oxidative stress [9, 12–14]. HO-1 gene product is responsible for heme catabolism that may result in CO production which subsequently increase gene expression of interleukin-10 (IL-10) and interleukin-1 receptors (IL-1R). This process can reduce inflammation [15]. Another gene that its expression increases oxidative stress is GADD45B. This increased expression affects stopping cell cycle survival, apoptosis, and DNA repair. Its expression pattern change reveals rate of cell damage at gene level [14].

Based on the important role of two mentioned genes in oxidative stress condition, the present study was designed to investigate the impact of the BPA on expression of HO-1 and GADD45B and clarify whether they can be considered as appropriate biomarkers following the BPA hepatotoxicity.

## 2. Materials and Methods

### 2.1. Chemicals and Reagents

Bisphenol A with purity >99% (Daejung, Korea), tripure and DEPC (Roche, Sigma, Germany), and C-DNA kit (Amplisens Co., Russia) were obtained. The primers for RT-PCR analysis were synthesized by Bioneer, Korea. All other chemicals were purchased from local commercial sources.

### 2.2. Animals

Male Wistar rats (10 to 12 weeks old) were prepared from animal room of Babol University of Medical Science (Babol, Iran).

All rats were housed in plastic cages under a well-regulated light and dark schedule (12 h light : 12 h dark) at 22 ± 2°C, at humidity (50 ± 5%) environment, and with free access to chow and tap water* ad libitum*.

The University Ethics Committee approved the study and all the experiments were performed in accordance with the guidelines for Care and Use of Laboratory Animals.

### 2.3. Treatment

The rats were randomly divided into four groups (*n* = 5). The BPA was given in three doses (5, 25, and 125 *μ*g/kg in corn oil as vehicle). It was orally administered every 24 hours for 35 days. The control group received olive oil alone.

### 2.4. Necropsy

All rats were fasted overnight after receiving the last dose of the BPA in corn oil. Then they were anesthetized using sodium thiopental and were finally sacrificed. Subsequently, the liver of each rat in specified group was removed and cleaned from adhering fat and connective tissues. The liver samples were quickly snap-frozen in liquid nitrogen and then were stored at −80°C for future experiments.

### 2.5. RNA Extraction

Total RNA was extracted from frozen rat liver tissues using tripure reagent kit and dissolved in diethylpyrocarbonate-treated deionized water; the quality and quantity of extracted RNA were assessed using Spectrophotometer (Thermo, USA) at 260 and 280 nm.

### 2.6. cDNA Synthesis and Real Time PCR Procedure

First-strand cDNA was synthesized by reverse transcription using Reverta-L RT Reagent kit, in accordance with the manufacturer's instruction. The thermocycler for cDNA synthesis was set up at 37°C for 30 min.

Quick PCR (qPCR) was performed using an Applied Biosystems 7300 Real Time PCR System (Applied Biosystems, Branchburg, NJ, USA) at three conditions, 95°C for 5 min, 45 cycles at 95°C for 30 s, and 60°C for 1 min. Expression levels of mRNA were normalized to* GAPDH *gene as the endogenous control. Then the relative differences between control and treatment groups were calculated and expressed as percentage of controls. Primers and probes for the qPCR were designed using Allele ID 6. All primers were listed in Table 1.

### 2.7. Statistical Analysis

All the analyses were performed using SPSS software version 19. Statistical analyses were performed using one-way ANOVA followed by* post hoc* Tukey test. The significance of differences between data was assumed at *p* < 0.05.

## 3. Result

### 3.1. Weight Changes

**Figure 1:**
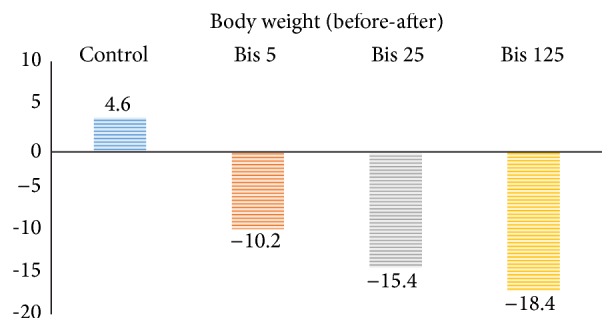
Mean of weight difference of baseline and end of treatment in control (olive oil) and bisphenol A receiving groups (*n* = 5). Bis 5: bisphenol A 5 *μ*g/kg, Bis 25: bisphenol 25 *μ*g/kg, and Bis 125: bisphenol 125 *μ*g/kg.

Body weight of the animals was recorded at the beginning of the experiment as baseline and before killing them. Based on the results, the weight was decreased in the BPA receiving groups compared to the control (Table 2 and Figure 1).

### 3.2. The Findings of Real Time PCR

**Figure 2:**
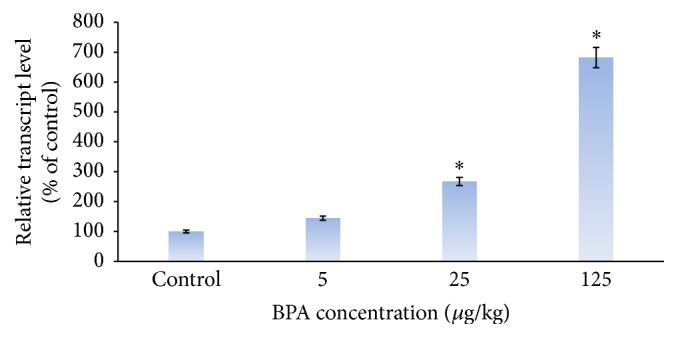
Dose-dependent increase in HO-1 gene expression in bisphenol A receiving groups compared to the control (olive oil). Higher effect is seen in dose of 125 *μ*g/kg bisphenol A receiving group. ^*∗*^ *p* < 0.05 significantly different from control group.

The results of Real Time PCR showed that different concentrations of the BPA increased the expression of HO-1 compared to the control group (*p* < 0.05). This increased expression was dose-dependent and there was a significant difference (*p* < 0.05) between the effects of medium dose (25 *μ*g/kg) and high dose (125 *μ*g/kg). Despite increased expression of HO-1, no significant difference was observed after administering dose of 5 *μ*g/kg BPA, compared to the control group (Figure 2).

**Figure 3:**
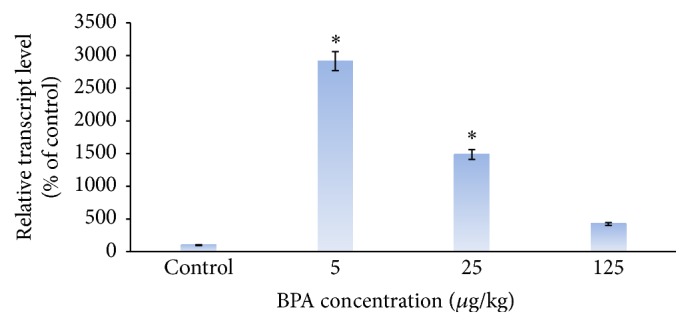
Dose-dependent increase in GADD45B gene expression in bisphenol A receiving groups compared to the control (olive oil). Higher effect is seen in dose of 5 *μ*g/kg bisphenol A receiving group. ^*∗*^ *p* < 0.05 significantly different from control group.

The results of Real Time PCR showed that administration of 5 and 25 *μ*g/kg doses of the BPA significantly increased GADD45B gene expression compared to the control (*p* < 0.05). This expression was significantly dominant in dose of 5 *μ*g/kg (Figure 3). Surprisingly enough, despite increase in expression of BPA concentration (25 and 125 *μ*g), the increased expression was not dependent on increased bisphenol A condensation; so gene expression was decreased at every level compared to previous level; and, contrary to GADD45B gene expression, the difference was not further significant at the concentration of 125 *μ*g of bisphenol A compared to the control group (Figure 3).

## 4. Discussion

The BPA is an estrogen-like chemical that can be released into the environment. The most studies have focused on its effects on reproductive system [16, 17]. The LD50 of the BPA (oral, rat) is 3.25 g/kg [18]. American environmental protection agency (EPA) has defined an acceptable daily dose of 50 *μ*g/kg of the BPA [19].

All three doses of the BPA induced a significant decrease in body weight in comparison to the control. Similar result was reported in a previous study on body weight loss [20].

It has been shown that the BPA by decreasing expression of the gene responsible for prevention of oxidative activity can induce production of ROS and subsequent hepatotoxicity [11].

HO-1 gene encodes an enzyme with the same name which can degrade heme molecule and help production of compounds such as carbon monoxide and biliverdin. In addition to these activities, continuous expression of the gene in steady state may be associated with adverse effects on living cells [21].

Although there are limited data on the effect of different concentrations of the BPA on gene expression in the liver, the present study in order to assess its liver cell toxicity showed a dose-dependent profile of increased gene expression of HO-1 as compared to the control. This expression of HO-1 gene was the highest in the group receiving largest dose of the BPA (125 *μ*g/kg).

Several investigations have focused on the role of the BPA in expression of genes and proliferation of cancer cells by stimulating the MAPK signaling pathway [22]. It has been shown that the BPA can affect the expression of Fkbp5 gene and its methylation, indicating its effective role on stress responses in cells [23].

The impact of the BPA on antioxidant activity of genes in the liver was also examined through Real Time PCR method. The results showed that the BPA causes the production of ROS which can significantly reduce the expression of antioxidant genes, leading to liver toxicity [24]. A study conducted on rats, which were exposed to doses of 0.1, 3, and 10 mM of inorganic arsenic for 72 hours, showed an increment in HO-1 gene expression [12]. It was also shown that HO-1 is a good biomarker to detect and define arsenic cell toxicity [25]. In another study on rats treated with various doses of the BPA (0.1, 1, 10, and 50 mg/kg), the compound led to changes in expression levels of antioxidant genes of glutathione, peroxidase (GSHPx), catalase (CAT), glutathione transferase (GST), and glutathione reductase (GR) in liver tissue. Expression of these antioxidant genes was decreased with increasing doses of the BPA. However, the impact of these doses of the BPA on biomarkers of oxidative stress showed a different result. With increasing doses of the BPA, the levels of TBARS and NO (*x*) were increased and GSH and SOD decreased [24].

Another result obtained in the present study showed that different doses of the BPA increased GADD45B gene expression compared to the control. This increased gene expression was significantly evident at the lowest doses (5 *μ*g/kg), but the gene expression was decreased in larger doses of the BPA compared to the low doses. Although an increased expression of GADD45B gene was shown in comparison to the control group, this increment was not statistically significantly the highest doses of the BPA. One of the results of this study is the positive effect of expression of this gene on cell apoptosis. When the rats received higher doses of the BPA, Gadd45 gene expression is increased first. Increased expression of Gadd45 gene stimulates apoptosis and the percent of viable cells for expression of this gene is being decreased gradually. As a result, with increasing doses of the BPA, gene expression followed a decreased trend. In the case of HO-1 gene, no study has reported changes in GADD45B gene expression after administering BPA in rats.

## 5. Conclusion

According to the results, it is concluded that bisphenol A (BPA) increased expression of HO-1 gene more than Gadd45 gene at high doses. The BPA can induce dose-dependent liver damage. Increased reactive oxygen species (ROS) and oxidative reactions may be responsible for the toxicity.

## Figures and Tables

**Table 1:** The primers and probes sequences for GAPDH, HO-1, and GADD45B genes.

Gene name	Forward primer (5′-3′)	Reverse primer (5′-3′)	FAM Probe
GAPDH	CTACATGGCCTCCAAGGAGTAAG	TGGAATTGTGAGGGAGATGCTC	ACCACCCAGCCCAGCAAGGATACT-TAMRA
HO-1	ACAGCATGTCCCAGGATTTGTC	GGAGGCCATCACCAGCTTAAAG	CCCTGGACACCTGACCCTTCTGAAAG-TAMRA
GADD45B	GAAGATGCAGGCGGTGACTG	CCTCCTCTTCTTCGTCTATGGC	CAGGCACAAGACCACGCTGTCGG-TAMRA

**Table 2:** Mean ± SD of the body weight of rat receiving the BPA and control.

Treatment groups	Body weight (g) at baseline	Body weight (g) after 35 days
Control	187 ± 8.72	191.60 ± 6.50
BPA (5 *µ*g/kg)	183 ± 9.95	172.8 ± 7.56^*∗*^
BPA (25 *µ*g/kg)	175.80 ± 9.01	160.4 ± 5.03^*∗*^
BPA (125 *µ*g/kg)	171.6 ± 6.68	168.0 ± 10.0^*∗*^

^*∗*^ *p* < 0.05 significantly different from control group.
